# Successful laparoscopy-assisted repair of a rectovaginal fistula after low anterior resection for rectal cancer: a report of two cases

**DOI:** 10.1186/s40792-021-01150-6

**Published:** 2021-03-16

**Authors:** Hiroyuki Ohta, Kyozo Hashimoto, Tomoyuki Mizukuro, Byonggu An, Yumi Zen, Yusuke Nishina, Yoshitaka Terada, Naomi Kitamura, Hiroya Akabori, Mitsuhiro Fujino, Eiji Mekata

**Affiliations:** 1 Department of Comprehensive Surgery, Shiga University of Medical Science, Seta-tsukinowa cho, Otsu, Shiga 520-2192 Japan; 2 Department of Surgery, Higashi-Ohmi General Medical Center, 255 Gochi-cho, Higashioumi, Shiga 527-8505 Japan; 3 Hashimoto Clinic, 2-9-12 Kaiden, Nagaokakyo, Kyoto 617-0826 Japan; 4 Nagaokakyo Hospital, 4-9-10 Kaiden, Nagaokakyo, Kyoto 617-0826 Japan

**Keywords:** Rectovaginal fistula, Rectal cancer, Low anterior resection, Double-stapling technique

## Abstract

**Background:**

Rectovaginal fistula (RVF) after low anterior resection for rectal cancer is troublesome and refractory. Although various surgical procedures have been previously described, no definitive procedure has shown a satisfactory outcome. We present two consecutive Japanese patients who underwent successful surgery for an RVF after low anterior resection.

**Case presentation:**

The patients were two women (61-year-old and a 64-year-old). They were admitted to our hospital with a chief complaint of fecal discharge from the vagina after low anterior resection using the double-stapling technique for rectal cancer. They were diagnosed with RVF. Local surgical procedures, including diverting ileostomy, were unsuccessful in previous hospitals. Therefore, we performed laparoscopy-assisted repair of the RVF. In both patients, laparoscopically robust pelvic adhesions were dissected, and the sigmoid colon was transected at just oral side to the RVF. Thereafter, in combination with a perineal approach, the rectum, along with a previous anastomosis and fistula, were completely removed. Surgeries were completed after vaginal repair, redo coloanal anastomosis, and interposition of the dissected connective tissue. In both patients, the postoperative courses were uneventful. They complained of neither recurrence of any RVF nor fecal incontinence 1 year and 10 months after diverting stoma closure.

**Conclusions:**

A laparoscopy-assisted procedure with reanastomosis and interposition of the perineal connective tissue can be an effective treatment for RVF after low anterior resection for rectal cancer.

## Background

Rectovaginal fistula (RVF), which is defined as the presence of an orifice between the rectum and the vagina, results in involuntary leakage of intestinal gas and stool from the fistula. Patients suffer from constant discomfort and psychological distress, which affects their quality of life. The most common cause of RVF in developing countries is obstetric vaginal trauma [1]. Low anterior resection (LAR) for rectal cancer using the double-stapling technique (DST) occasionally leads to RVF in developed countries, with a reported incidence of 1.0–9.9% [2–5].

Various surgical techniques such as temporary diverting stoma, endorectal advancement flap [6], and gracilis muscle interpositions [7] have been previously described in the literature. However, the optimal surgical treatment for RVF remains controversial [8]. Here, we report two patients who underwent successful laparoscopy-assisted repair of RVF after LAR for rectal cancer.

## Case presentation

### Patient 1

**Fig. 1:**
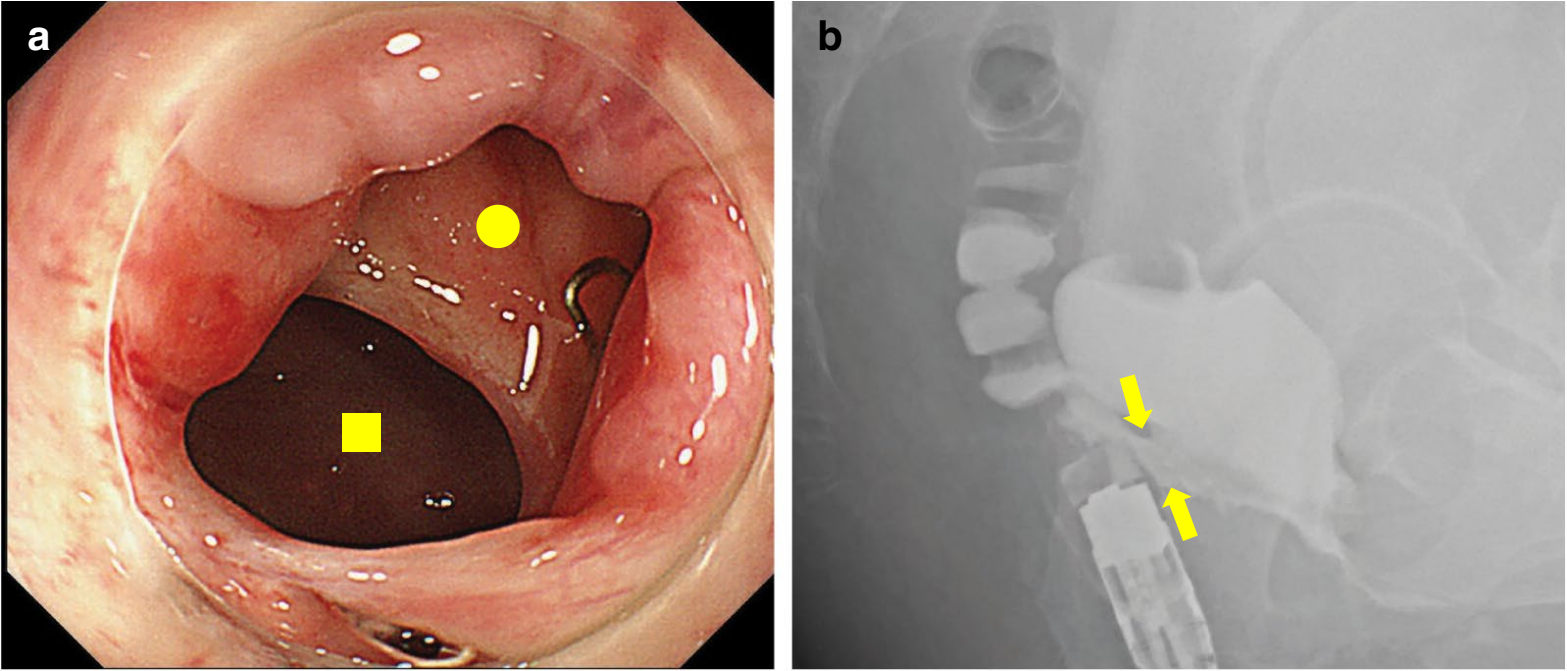
**a** Colonoscopic examination demonstrates a rectovaginal fistula 10 mm in diameter, located 3 cm from the anal verge, on the oral side. Closed circle: rectal lumen, Closed square: vaginal lumen. **b** Gastrografin enema shows influx of the contrast medium to the vagina through a fistula (arrows)

**Fig. 2:**
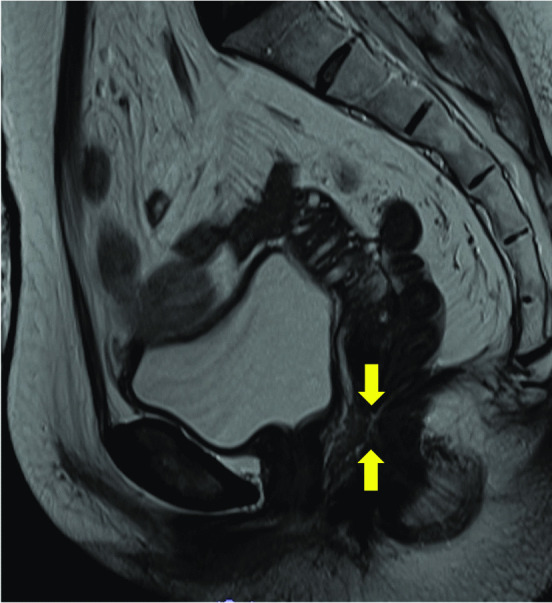
Magnetic resonance imaging shows a fistula between the rectum and vagina (arrows). No pelvic abscesses or recurrent tumors are seen

A 61-year-old Japanese woman was admitted to our hospital with a chief complaint of fecal discharge from the vagina. She received laparoscopic LAR using the DST for early disease-stage rectal cancer 2 years and 9 months ago in another hospital. Surgical site drainage and diverting ileostomy were also performed due to anastomotic leakage on the 4th day after surgery. Anastomotic leakage seemed to have healed 4 months after ileostomy; therefore, stoma reversal was performed accordingly. Immediately after surgery, she noticed fecal discharge from the vagina and was diagnosed with an RVF. Despite a second diverting ileostomy and transvaginal repair, the RVF had not cured completely. Colonoscopic examination revealed an RVF measuring 10 mm in diameter, situated 3 cm from the anal verge, on the oral side (Fig. 1a). Gastrografin enema showed an influx of contrast medium into the vagina (Fig. 1b). Magnetic resonance imaging showed a fistula between the rectum and the vagina. Pelvic abscesses or recurrent tumors were not observed in the patient (Fig. 2).

Local minor surgical procedures, including diverting ileostomy, were unsuccessful; therefore, we thought that a major procedure with coloanal reanastomosis was necessary. Laparoscopy-assisted repair of the RVF was performed under general anesthesia with the patient in the lithotomy position. The procedure consisted of three steps.

The first step involved careful dissection of the sigmoid colon and retroperitoneal tissue, to avoid injury to the mesocolonic vessels. Mobilization of the splenic flexure was then performed to decrease tension on the coloanal anastomosis. In the pelvic cavity, the sigmoid colon was detached from the anterior surface of the sacrum, and adhesions around the uterus next to the RVF were removed. Intracorporeal sigmoid colon transection was performed with a linear stapler on the oral side of the RVF.

**Fig. 3:**
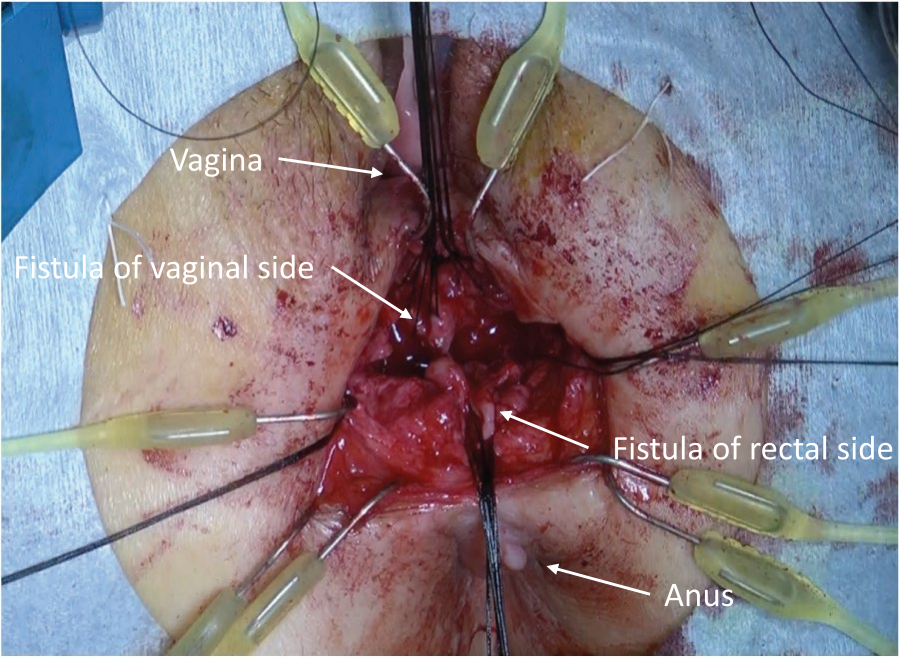
During the dissection of connective tissues between the rectum and vagina, a rectovaginal fistula was released completely

**Fig. 4:**
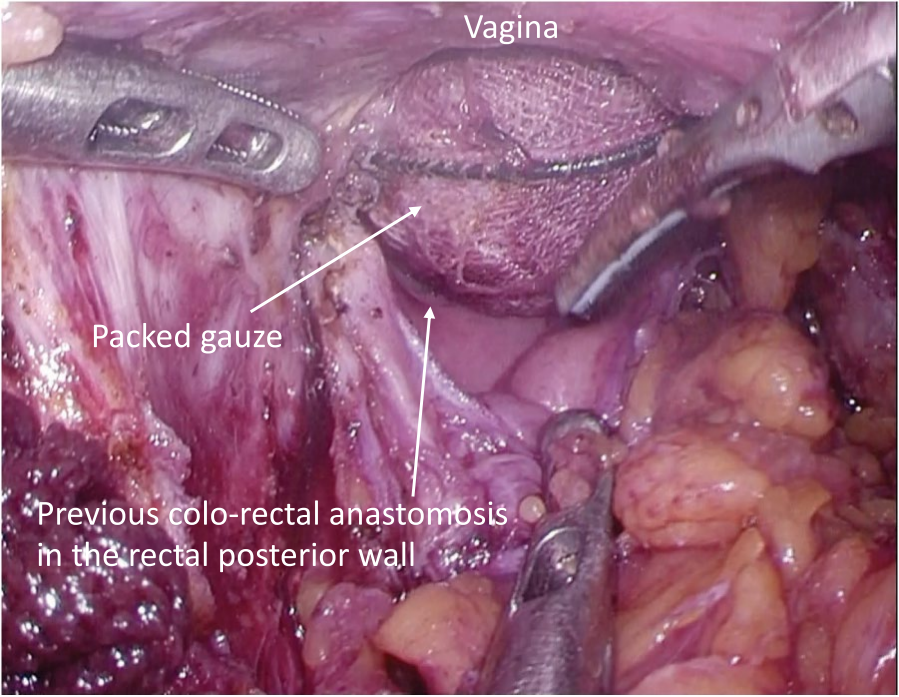
While resectioning the rectum containing previous anastomosis, a moist gauze was packed in opened perineal space for maintaining pneumoperitoneum

**Fig. 5:**
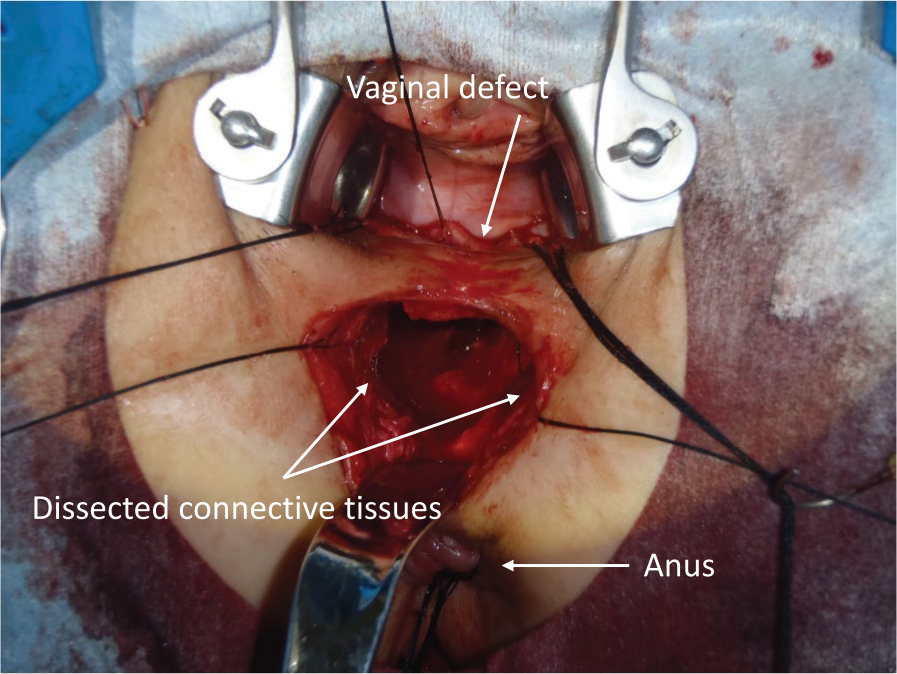
After the complete resection of the previous anastomosis and RVF, dissected connective tissues were exposed

In the second step, a transverse skin incision was made on the perineum. Subcutaneous dissection was performed carefully to avoid injury to the outer margin of the external anal sphincter. The connective tissues between the rectum and the vagina were dissected up to a depth of approximately 6 cm from the skin level. During this dissection, the fistula was released (Fig. 3), and the surrounding infectious tissue was removed completely. The fibrous connective tissues around the RVF were clearly exposed on each lateral side. In addition, using the laparoscopic approach, the sigmoid colon, including the previous anastomosis, was transected and removed at the level of the superior border of the puborectal sling (Fig. 4). The remnant mucosa of the anal canal has been transanally removed at the level of the dentate line. Figure 5 shows the intraoperative findings after the complete resection of the previous anastomosis and RVF.

**Fig. 6:**
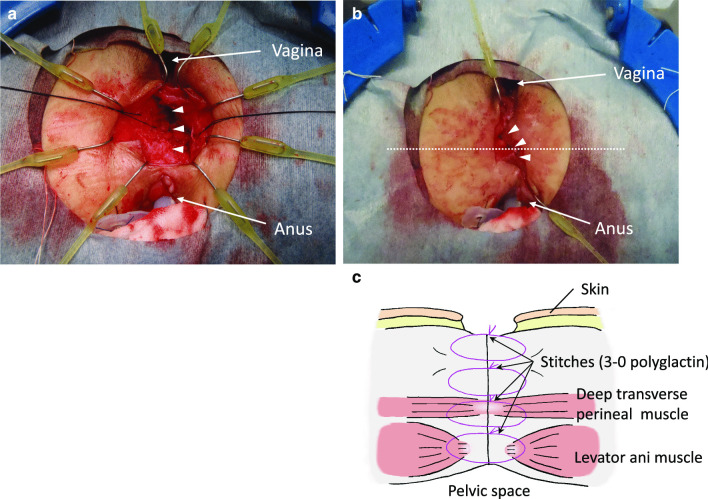
**a** Innermost portion of the dissected connective tissue was sutured longitudinally by making three stitches (arrow heads). **b** After the repair and interposition of the dissected connective tissue. The outermost layer was also sutured by making three stitches (arrow heads). **c** Illustration at the cross section of the dotted line in **b**

In the third step, the defect on the posterior vaginal wall was closed using interrupted 3–0 polyglactin sutures. Subsequently, redo coloanal anastomosis was performed transanally using the Gambee suture pattern with the hand-sewn technique. We put 18 stitches using interrupted 3–0 polyglactin sutures, and a drainage tube was inserted into the intestinal lumen to decompress the anastomotic site. Furthermore, firm longitudinal suturing of the innermost portion of the dissected fibrous tissues around the RVF on each of the lateral sides was performed by making three stitches using 3–0 polyglactin (Fig. 6a). In the same way, more superficial dissected tissues were repaired in four layers from bottom to top through three stitches per layer, and the repaired tissues were interposed between the vagina and the neo-rectum including the redo coloanal anastomosis (Fig. 6b). These layers were difficult to describe in definite anatomical terms; however, the innermost portion supposedly contained the levator ani muscle. The surgical procedure of perineal reconstruction before skin closure is depicted in Fig. 6c, which is illustrated in the cross section of the dotted line in Fig. 6b. The operation lasted for 691 min, and the estimated blood loss was 795 g.

The postoperative clinical course was good, and the patient was discharged from the hospital on the 16th postoperative day. Stoma closure was performed 11 months after surgery. She had no recurrence of RVF 1 year after diverting stoma closure. Regarding defecation function, she sometimes had passive fecal incontinence to liquid stool up to 6 months after stoma closure, but she had no complaint of fecal incontinence 1 year after surgery. Nevertheless, she regularly wore pads due to anxiety about incontinence.

### Patient 2

A 64-year-old Japanese woman was referred to our hospital with a chief complaint of fecal discharge from the vagina. She received laparoscopic LAR using the DST for early disease-stage rectal cancer 1 year prior to her referral. On the 3^rd^ day after surgery, she complained of fecal discharge and flatulence from the vagina and was diagnosed with an RVF. Diverting ileostomy was performed 8 months prior to her referral, but the RVF had not cured completely. A fistula measuring approximately 2 mm in size was found 4.5 cm from the anal verge on the oral side.

Laparoscopy-assisted repair of the RVF was performed in the same way as in the previous patient. The operation lasted 615 min, and the estimated blood loss was 700 g. The postoperative clinical course was uneventful. She had no recurrence of RVF 10 months after stoma closure. Regarding defecation function, she sometimes had passive fecal incontinence to liquid stool up to 3 months after stoma closure, but she had no complaint of fecal incontinence 6 months after surgery. Nevertheless, she also regularly wore pads due to anxiety about incontinence, a similar observation for the previous patient.

## Discussion

Anastomotic healing disturbances following LAR occasionally lead to RVF. Possible etiologies of RVF include the following: stapling of the vagina during anastomosis, spontaneous drainage of a pelvic abscess through the vagina, and devascularization of the vagina near the anastomosis [9]. There are two approaches for managing RVFs: local conservative procedures (rectal or vaginal advancement flaps, fistula plug, fibrin glue, and diverting stoma only) and more aggressive procedures (gracilis muscle interposition and abdominal surgery with colorectal or coloanal reanastomosis) [10]. Recent reports suggest that aggressive surgical treatment of RVF, including early construction of a diverting stoma and abdominal surgery with redo anastomosis, might result in high success rates [10, 11]. However, abdominal surgery with redo anastomosis could be technically troublesome because of previous anastomotic disturbance and consequent severe pelvic adhesion. In our experience, laparoscopy was helpful for adequate dissection of the vagina and rectum in patients who underwent previous surgery of the pelvic cavity. To the best of our knowledge, there are no reports of laparoscopic treatment with redo anastomosis for RVF after LAR in the English literature.

A unique feature of our procedure is the repair and interposition of the dissected connective tissue between the anastomosed vagina and colon. We cannot describe the dissected connective tissue in anatomical terms accurately, although the levator ani muscle and deep transverse perineal muscle might be resutured partially. Tissue interposition approaches, such as gracilis muscle interposition or omental interposition, are intended to interpose well-vascularized tissues between suture lines and contribute to wound healing [7, 12]. However, in gracilis muscle interposition, a large skin incision is required on the thigh, and postoperative motility disturbance can potentially occur. Meanwhile, a pedicled omental flap may not be pulled down the perineum occasionally due to severe adhesion or lack of tissue. In our procedure, we think that perineal connective tissue may not be well-vascularized but it possesses enough elasticity to fill the dissected perineal space. Thus, there is the advantage of avoiding another skin incision and not being affected by the adhesional status of the omentum, compared to other interposition approaches.

In our case, the location of the RVF was low and near the vaginal fourchette. Therefore, a perineal approach with fistulectomy and interposition of the dissected perineal connective tissue was possible. When the location of an RVF is high, the only abdominal approach with fistulectomy and colorectal reanastomosis by the DST using a stapling device could have been sufficient. Furthermore, additional omental interposition should also be considered to promote wound healing.

There is no conclusive evidence of the long-term efficacy of our method. Therefore, similar cases should be followed for a longer time after surgery.

## Conclusion

We successfully performed two consecutive surgeries in patients with RVF after LAR for rectal cancer. Laparoscopy-assisted procedures with reanastomosis and interposition of the dissected perineal connective tissue may improve outcomes in patients with RVF.

## Data Availability

We will share our data and materials on demand.

## Abbreviations

RVF: Rectovaginal fistula
LAR: Low anterior resection
DST: Double stapling technique
